# The critical role of surface dipoles in CsPbI_3_ perovskite solar cells

**DOI:** 10.1039/d5ee07787g

**Published:** 2026-03-11

**Authors:** Ran Ji, Nathaniel Gallop, Shivam Singh, Richard Beier, Yitian Du, Zongbao Zhang, Fulya Koc, Marielle Deconinck, Vladimir Shilovskikh, Jose Roberto Bautista-Quijano, Boris Rivkin, Yana Vaynzof

**Affiliations:** a Chair for Emerging Electronic Technologies, Technical University of Dresden Nöthnitzer Str. 61 01187 Dresden Germany; b Leibniz-Institute for Solid State and Materials Research Dresden Helmholtzstraße 20 01069 Dresden Germany y.vaynzof@ifw-dresden.de

## Abstract

Interfacial modification is a key strategy for improving the performance of perovskite photovoltaic devices. However, most commonly, improvements in device performance through surface treatments of the perovskite active layer are attributed to defect passivation. At the same time, such treatments may also lead to the formation of a dipole at the surface of the perovskite active layer. In this work, we demonstrate that treatments that modify the surface stoichiometry of CsPbI_3_ perovskites can lead to effective defect passivation, yet result in the formation of surface dipoles of opposing directions that modulate the work function of CsPbI_3_ over a range of more than 2 eV. Such dipoles influence the built-in potential of the devices and the efficiency of interfacial charge transfer in CsPbI_3_ solar cells, resulting in power conversion efficiencies that increase from below 10% to ∼20% depending on the surface dipole. The surface stoichiometry also has a strong influence on device stability, where initially high-performing devices are found to be prone to a more rapid degradation. These results highlight that the formation of surface dipoles plays a crucial role in impacting the performance and stability of CsPbI_3_ solar cells, making the choice of device architecture particularly important.

## Abstract 2

Finely modulating the Cs : Pb ratio of the top 1 nm of CsPbI_3_ leads to strong surface dipoles that alter the work function by more than 2 eV, increasing the power conversion efficiency from ∼10% to ∼20% and critically affecting stability.

## Graphical abstract

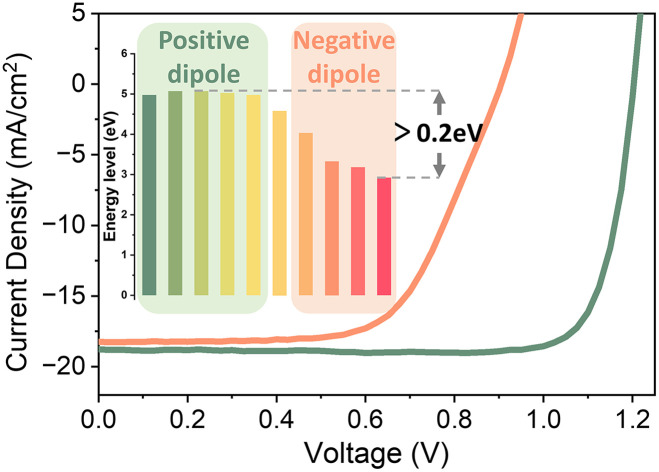

**Broader context**

The all-inorganic CsPbI_3_ perovskite composition has garnered increasing attention due to its wide bandgap (∼1.68 eV), simple halide composition, and excellent thermal stability. When processed from solution, most reports rely on the so-called “dimethylammonium iodide (DMAI) method,” as it enables the formation of a black perovskite phase at moderate temperatures. However, the passivation of CsPbI_3_ perovskite surfaces does not always lead to improved device performance, suggesting that other, inconspicuous mechanisms are at play. In this work, we demonstrate that CsPbI_3_ films fabricated *via* the DMAI method intrinsically possess a Cs-rich surface. Finely adjusting the Cs : Pb elemental ratio within the top 1 nm surface region yields the formation of strong surface dipoles, which, depending on their polarity, can either enhance or diminish device performance, despite the surface being well passivated in both cases. These results reveal that the formation of surface dipoles plays a crucial role in determining the performance of CsPbI_3_ perovskite solar cells, providing insights into interfacial design and device architecture selection.

## Introduction

Perovskite solar cells (PSCs) have developed rapidly in recent years, achieving certified stabilized power conversion efficiencies (PCEs) of 27%.^1^ A key factor driving this advancement is the development of surface modification methods, including the use of low-dimensional perovskites, aromatic amines, and ammonium ligands.^2–8^ It is generally considered that the primary mechanism through which interfacial modification leads to improved device performance is the passivation of defects, which would otherwise induce surface recombination by trapping carriers.

Most passivation strategies target the most common defect—the halide vacancy—which has a low formation energy.^9,10^ For example, You *et al.* performed PEAI surface treatment on perovskite films in devices with a standard structure, enabling the open-circuit voltage (*V*_OC_) to reach 94.4% of the Shockley limit, surpassing that of silicon-based solar cells.^7^ More recently, Kanatzidis *et al.* developed a series of perovskitoid spacers based on two-dimensional (2D) spacer passivation. These spacers have more robust octahedral networks and contain longer ammonium groups, which can suppress cation diffusion while effectively passivating defects, thus enhancing the performance and stability of solar cells.^11^ These and other examples demonstrate that interfacial passivation strategies are highly effective in improving the performance of perovskite solar cells. Oftentimes, however, surface treatment methods reported in the literature for NIP-structured devices do not apply to PIN-structured devices.^7,12–14^ This is surprising, since defect passivation should lead to a performance increase regardless of device architecture. This suggests that, in addition to defect passivation, such surface treatments may also induce an interfacial electric field in a specific direction, thereby only yielding significant performance gains for either standard or inverted structures.

The modulation of the interfacial electric field can significantly impact device performance due to the existence of near-interface minority carriers that may directly recombine with the majority carriers—a process that can occur even at non-defective sites.^15^ While both defect passivation and interfacial electric fields can have a strong influence on the device performance, it is challenging to elucidate the distinct contributions of these two mechanisms.

One possible strategy to modulate the perovskite surface electric field is to dope the surface of the perovskite film, thereby reducing the surface minority carrier concentration. Various organic molecular dopants have been applied to the perovskite surface for this purpose. For instance, Wang *et al.* achieved n-type charge transfer doping on the perovskite surface using benzyl viologen in an inverted architecture, which reduced the hole minority carrier concentration and increased the device *V*_OC_ by 70 mV.^16^ In contrast, Huang *et al.* implemented p-type doping using the small organic molecule PT-TPA in a standard architecture, which increased the conductivity and carrier concentration of perovskite by a factor of approximately 4700 and raised the device *V*_OC_ from 1.12 V to 1.17 V.^17^ However, the photovoltage changes induced by doping in the aforementioned studies were relatively limited.

Alternatively, the surface electric field can be modulated by introducing molecular dipoles at the surface of the perovskite layer. For example, Wong *et al.*, introduced different derivatives of dicationic phosphonium-bridged ladder stilbenes to modulate the work function (WF) of the perovskite layer by ∼0.35 eV, which led to improved device performance only if the energetic level alignment of the derivatives was beneficial for charge extraction.^18^ Recently, molecular dipoles have also been used by Ullah *et al.* to improve the performance of PIN devices.^19^ Sargent *et al.* further combined defect passivation with surface electric field modulation and reported a performance improvement from 23% to 26%.^20^ These examples combine defect passivation and interfacial electric field modulation. Yet, the resultant change in *V*_OC_ is only on the order of 20–35 mV, indicating that the ability to modulate the surface electric field using diamine ligands is limited.

In this work, we demonstrate that surface modifications in inorganic CsPbI_3_ perovskites result in significant variations of the surface electric field, leading to a change in the surface WF over a broad range of 2 eV, producing both positive and negative dipoles at the surface of CsPbI_3_. Modifications with opposing dipole directions lead to a change in the *V*_OC_ of the devices from 0.8 V to 1.2 V, despite the surface being well passivated by either modification. This work elucidates the effects of defect passivation and electric field modulation on device performance, offering new insights into the formation mechanism of surface dipoles in CsPbI_3_ perovskite and their crucial role in determining the efficiency and stability of solar cells.

## Result and discussion

### Understanding the surface of CsPbI_3_ prepared *via* the dimethylammonium iodide (DMAI) method

The most common method for depositing CsPbI_3_ layers by solution processing is *via* the “DMAI method”.^21–27^ DMAI is highly effective as an additive for fabricating CsPbI_3_ films due to its matching crystallization rate with CsI, the compliance of ion radii with the Goldschmidt tolerance factor, and volatility. The latter makes it possible to eliminate DMAI from the perovskite layer upon an annealing step at temperatures above 180 °C.^28–30^ This high temperature is required to crystallize CsPbI_3_ in its β-phase.

To characterise the surface composition of the films, X-ray photoemission spectroscopy (XPS) measurements were used to evaluate the element ratios of films prepared using different annealing conditions (Fig. S1). The I : Pb ratio at the surface of the unannealed film was found to be 5.7, and the Cs : Pb ratio reached 2.3—both far exceeding the stoichiometry of PbI_2_ and CsI that were added at a ratio of 1 : 1 to the solvent. Upon annealing at 180 °C, the I : Pb ratio decreased to approximately 4.2, and the Cs : Pb ratio decreased to 1.7, still substantially higher than the stoichiometry in the solution. Further increasing the annealing temperature to 210 °C, the I : Pb ratio and Cs : Pb ratio continued to decrease with increasing annealing temperature and duration; however, the change in stoichiometry was slight, with the I : Pb and Cs : Pb ratios stabilizing at approximately 4 and 1.5, respectively, indicating that CsPbI_3_ films prepared using the DMAI recipe have an intrinsic CsI-rich surface. This may be due to the sequential precipitation of precursors resulting from their differing solubilities. Although increasing the annealing temperature can partly alleviate the imbalance in element ratio at the CsPbI_3_ surface, it does not enable the attainment of a perfect stoichiometric ratio—possibly due to the limited ion diffusion range in the solid film. Considering that high annealing temperatures had only a mild effect on the surface stoichiometry, but are detrimental to the stability of the MeO-2PACz layers used as hole transporting layers (HTLs),^31^ the annealing temperature was kept at 180 °C.

### Altering the stoichiometry of CsPbI_3_ films and the effect on photovoltaic devices

**Fig. 1:**
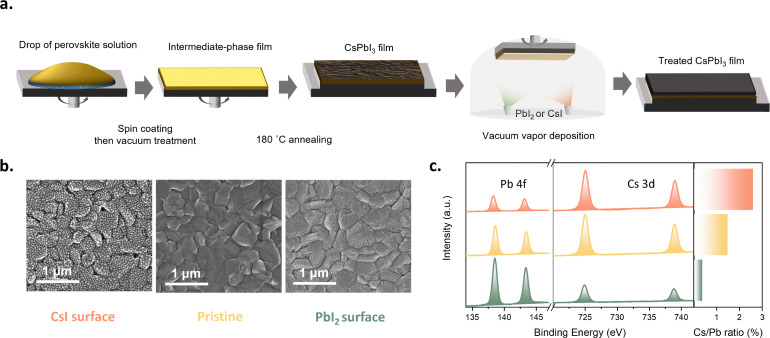
(a) Schematic diagram of the surface treatment method (b) SEM images (c) XPS of treated surfaces and Cs : Pb element ratios.

**Fig. 2:**
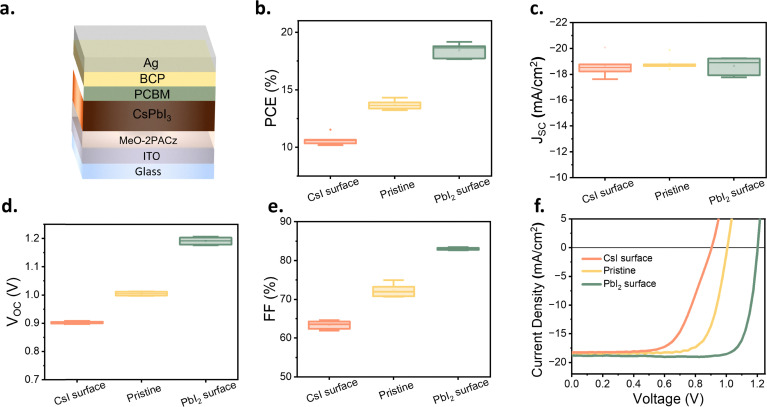
Photovoltaic performance of treated CsPbI_3_. (a) Architecture of PV device. (b) PCE distribution, (c) *J*_SC_ distribution, (d) *V*_OC_ distribution, (e) FF distribution, (f) *J*–*V* characteristics of treated CsPbI_3_ solar cells. The box plots in panels (b)–(e) display the mean, median line, 25%–75% box limits with 1.5× interquartile range whiskers. The total number of samples is 18. The corresponding values in panels (b)–(e) are provided in the Table S1. The forward and reverse scans of f are provided in the Fig. S3.

Numerous studies have shown that adjusting the ratio of MAI to PbI_2_ can induce significant self-doping effects in MAPbI_3_.^32^ For CsPbI_3_ perovskite, excess CsI in CsPbI_3_ may alter the crystal structure of CsPbI_3_: CsI splits [PbI_6_]^4−^ into isolated octahedral structures, leading to a phase transition from 3D to 0D.^33^ Therefore, it is reasonable to assume that an imbalance in surface element ratio may cause changes in CsPbI_3_ properties. To accurately control the surface stoichiometry of the perovskite layer and explore its impact on perovskite properties, we developed a combined method of solution processing and evaporation, as shown in Fig. 1a. The solution processing step adopts the aforementioned DMAI recipe; subsequently, the solution-processed CsPbI_3_ film is transferred to an evaporation chamber. Evaporating 1 nm of CsI or PbI_2_ strongly modulates the surface element ratio of CsPbI_3_. It is worth noting that the 1 nm thickness here is measured using a quartz crystal microbalance (QCM), as it is difficult to form a continuous film of this thickness *via* evaporation, making direct thickness measurement challenging. Fig. 1b shows the surface scanning electron microscopy (SEM) images of the films as cast and after evaporating 1 nm CsI or PbI_2_. In the following, these three conditions are abbreviated as “CsI surface,” “pristine,” and “PbI_2_ surface,” respectively. Compared with the surface of the as-cast films, the CsI surface maintained the original size of perovskite crystals but exhibited nanoscale island structures—reasonably attributed to the evaporated CsI. The discontinuous morphology is also consistent with our expectation of an ultra-thin layer. In contrast, the PbI_2_ surface exhibited a relatively indistinct nanostructured morphology with lower contrast compared to the CsI surface, possibly due to soft smoothing of the surface by a thin continuous PbI_2_-enriched layer. Fig. 1c presents the surface XPS spectra of the three types of samples. The results indicate that the surface Cs : Pb ratio can be significantly tuned from 0.4 to 2.7 using this strategy, allowing us to modulate the surface stoichiometry substantially. Notably, the bulk properties of the CsPbI_3_ layer remain unaffected, as is evidenced by X-ray diffraction (XRD, Fig. S2a) and ultraviolet-visible spectroscopy (UV-Vis, Fig. S2b) measurements. Moreover, surface-sensitive grazing incidence X-ray diffraction (GIXRD, Fig. S2c) measurements suggest that the change in surface stoichiometry is not accompanied by a change in crystal structure. To examine the impact of surface stoichiometry on the photovoltaic performance of the devices, the layers were integrated into inverted architecture devices with the architecture ITO/MeO-2PACz/CsPbI_3_/PC_61_BM/BCP/Ag (Fig. 2a), which follows our previously published work.^34^

The PCEs of solar cells with different surface Cs : Pb element ratios show a significant difference (Fig. 2b): the PCE increased from ∼10% (CsI surface) to nearly 20% (PbI_2_ surface). Interestingly, Fig. 2c shows essentially no variation in the device short-circuit current density (*J*_SC_), with the current density ranging from 18 mA cm^−2^ to 19 mA cm^−2^, regardless of the layer surface stoichiometry. In contrast, the device *V*_OC_ was significantly affected by the surface Cs : Pb element ratio, varying from 0.9 V (CsI surface) to 1.2 V (PbI_2_ surface).

This change was accompanied by a similar evolution of the fill factor (FF), which increased from 60%–65% (CsI surface) to 83–84% for devices with a PbI_2_ surface. The trend is further visualized in Fig. 2f, which displays the *J*–*V* curves of devices with different surface types. While the short current density remains unaffected, the change in stoichiometry from CsI surface towards PbI_2_ surface leads to a substantial increase in the *V*_OC_ and FF.

### Impact of surface stoichiometry on recombination and charge extraction at the CsPbI_3_/PCBM interface

**Fig. 3:**
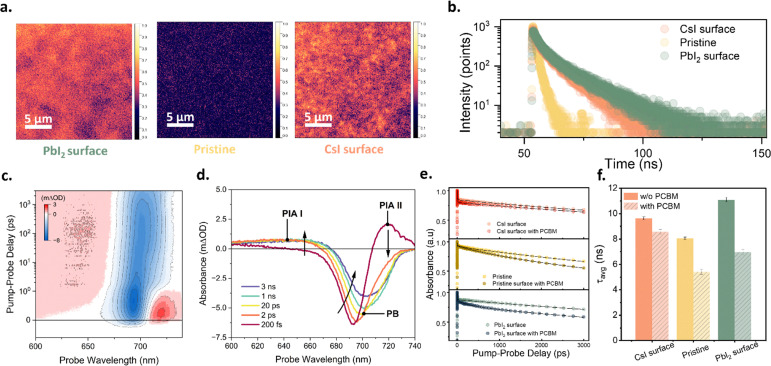
(a) PL mapping and (b) TRPL intensity of treated CsPbI_3_ films. (c)–(f) Carrier dynamics in CsPbI_3_ as determined *via* femtosecond transient absorption. (c) 2D femtosecond transient-absorption spectroscopy map of pristine CsPbI_3_ and; (d) spectral slices of (c) at exponentially increasing pump–probe delays. The prominent excited state absorption (PIA I and PIA II) and ground state bleach (GSB) features are labelled with arrows, as is the observed spectral diffusion of the GSB on picosecond timescales. (e) Carrier population kinetics as extracted *via* SVD. The solid black lines represent the exponential fits for each system. (f) Corresponding average lifetimes for each kinetic. The colors in (a) only indicate the PL intensity and do not correspond to the actual PL emission colors.

To understand the mechanism responsible for the significant changes in device performance caused by adjustments to the surface element ratio, the samples were characterised using photoluminescence (PL) microscopy (Fig. 3a). Both the PbI_2_ surface and CsI surface exhibited stronger PL intensities—of similar magnitude—compared with the pristine sample. Furthermore, we characterised the time-resolved photoluminescence (TRPL) of these samples (Fig. 3b). The PL lifetimes of the PbI_2_ surface and CsI surface samples were similar and substantially longer than that of the pristine sample. The enhanced PL intensity and extended PL lifetime in Fig. 3a and b confirm that both the PbI_2_ and CsI surface samples exhibit reduced non-radiative recombination than the pristine sample, indicating that both PbI_2_ and CsI passivate and reduce the surface defects of CsPbI_3_ films.

To examine charge-carrier recombination behavior under typical solar cell operating conditions, PL spectra of complete solar cell devices were measured under 1 sun excitation (Fig. S4). Devices with CsI- and PbI_2_-modified surfaces both exhibit higher PL intensities than the pristine device. Assuming comparable light-in-coupling and light-out-coupling efficiencies across all three samples, we attribute the increased PL intensity primarily to changes in the radiative recombination coefficient, which, in agreement with the PL microscopy measurements, confirms that both CsI and PbI_2_ surface treatments effectively suppress non-radiative recombination compared to the pristine surface. However, this phenomenon is surprising in light of the significant difference in *V*_OC_ among devices with different surface Cs : Pb ratios. Thus, we hypothesize that the passivation of surface defects is not the decisive factor causing the substantial *V*_OC_ variation in devices with different surface Cs : Pb ratios.

To further analyse the charge carrier dynamics in the samples and across the interface to the PC_61_BM electron transport layer, we employed femtosecond transient absorption (TA) spectroscopy. We excited the perovskites at a wavelength of 400 nm with a fluence of 1.8 µJ cm^−2^. Fig. 3c displays an exemplary TA map of CsPbI_3_, along with spectral cuts at various pump–probe delay times. The response is dominated by a ground-state bleach (GSB) feature at approximately 700–720 nm, accompanied by two excited-state absorption (ESA) signals (labelled PIA I and PIA II in Fig. 3d). The ESA II signal below the bandgap becomes more prominent under above-bandgap excitation and is attributed to the relaxation of hot carriers. In general, the TA spectra of hybrid perovskite materials comprise many overlapping contributions from a variety of effects, including the Burnstien–Moss effect,^35^ bandgap renormalisation, refractive index changes, alongside the previously discussed hot-carrier and ground state bleach signals discussed previously.^36^ The transient nature of some of these effects (in particular, hot carrier effects) leads to the spectral diffusion observed in Fig. 3c, where the ground state bleach feature undergoes a *ca.* 20 nm redshift on timescales of 1–10 ps. These effects can make the extraction of charge carrier dynamics from the analysis of single-wavelength kinetics difficult, necessitating alternative approaches. To this end, we employed two-component singular value decomposition (SVD) to separate the bare carrier population dynamics from these other effects. The as-extracted spectral profiles and kinetics of the two SVD components obtained from the 2D TA map given in Fig. 3c are shown in Fig. S3a and b, respectively. The first of the two components exhibits a spectral profile comprising a prominent bleach feature centred at the band edge, as well as weaker excited-state absorption (ESA I). The spectral shape and temporal behaviour of this component align well with signals associated with an excited-state population; therefore, we interpret this component as the spectral signature of the carrier population within the perovskite film. Contrastingly, Component 2 exhibits a derivative-like spectral profile, indicative of spectral diffusion. Moreover, the complicated kinetic profile of component 2—an initial positive spike followed by a rapid decay to an overall negative value—suggests that it does not represent a stable, long-lived carrier population. Instead, we assign component 2 to a mixture of transient energetic redistribution processes, including hot carrier cooling and band renormalization. As our primary interest in this study is the population dynamics of carriers within our perovskite systems, we do not analyse component 2 further in the following discussion, focusing instead on charge carrier behaviour.

We quantify our obtained charge carrier dynamics by fitting them with a phenomenological three-exponential model convoluted with a 150 fs instrument response function. We extract the amplitude-weighted lifetime (*τ*_avg_) for each kinetic using the equation:
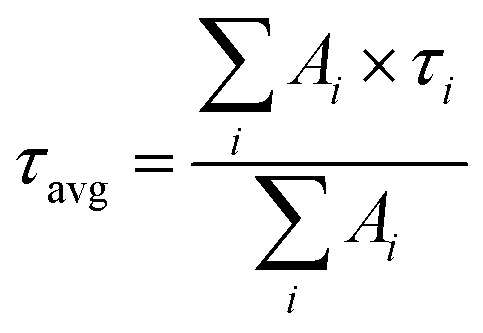
where *A*_*i*_ and *τ*_*i*_ is respectively the amplitude and the lifetime of the *i*-th component of the exponential decay. While we acknowledge that other models, such as the ABC, Shockley–Reed–Hall, and SRH+ models, more accurately reflect the actual carrier dynamics within hybrid perovskite,^37,38^ we aim to quantify the overall mean lifetime of the carrier population, making the relatively straightforward exponential model sufficient.

Further, we characterised samples with different surface Cs : Pb ratios. For each interface, we obtained charge carrier dynamics for both the bare perovskite and the same system interfaced with a PC_61_BM electron transport layer (ETL). The resulting carrier population kinetics for the three systems under investigation (pristine, PbI_2_ surface, CsI surface)—both with and without a PCBM interface—are shown in Fig. 3e, while the obtained average lifetimes of each transfer from the perovskite to the ETL. We further quantified this by calculating the difference in average lifetime between the bare perovskite and the corresponding perovskite/PC_61_BM bilayer system. This difference is ascribed to the presence of an additional charge transfer pathway, with larger lifetime changes corresponding to more efficient charge transfer. These results are summarized in Table S1. Interestingly, we found that while PbI_2_ surface enhanced charge transfer relative to the pristine sample, the opposite was true for CsI surface perovskite films. Thus, although both CsI and PbI_2_ treatments passivate the perovskite, CsI surface actively inhibits charge transfer, while PbI_2_ promotes it. This finding is in good agreement with the observed PCE trend of the corresponding PbI_2_ surface, pristine, and CsI surface devices summarized in Fig. 2.

### Impact of surface stoichiometry on the energetic landscape and device performance

The observation of effective surface defect passivation, along with opposing trends in the efficacy of charge extraction between the PbI_2_ and CsI surfaces, suggests that the surface electric field differs between the two types of surfaces. To quantify this difference, we characterised the surface WF of CsPbI_3_ modified by different means to regulate the surface stoichiometry *via* ultraviolet photoemission spectroscopy (UPS). To explore the impact of surface stoichiometry more broadly, we characterised not only the PbI_2_ and CsI surface samples, but also samples modified with other metal halides (PbBr_2_, PbCl_2_, CsBr, CsF, RbI) as well as other methods reported to alter surface stoichiometry, such as 1,4-butanediamine (DAB) and methanol treatments,^39,40^ that were reported to dissolve CsI, leading to a reduction in the surface Cs : Pb ratio.

**Fig. 4:**
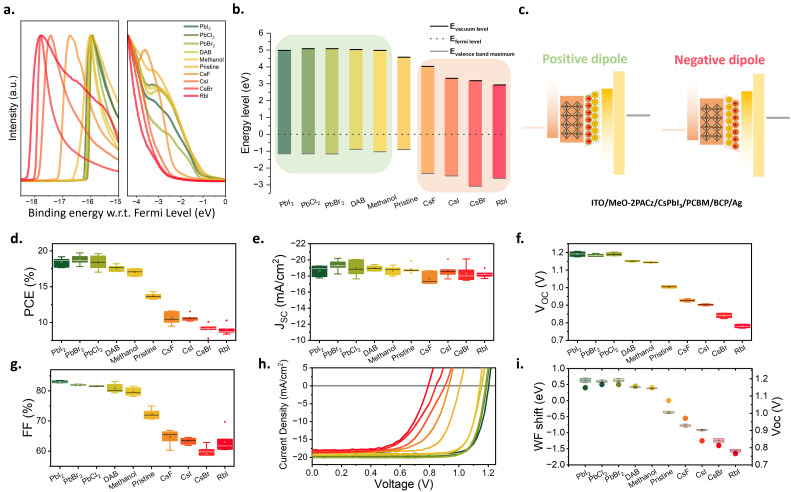
(a) UPS spectra of CsPbI_3_ with different surface treatments, where the colour of the curves and the corresponding surface treatment conditions are consistent with those in panel (b). (b) Energy level diagram obtained from (a). (c) Schematic illustration of the dipole formed between CsPbI_3_/PCBM. (d)–(h) Photovoltaic performance of treated CsPbI_3_: (d) PCE distribution (e) *J*_SC_ distribution (f) *V*_OC_ distribution (g) FF distribution (h) *J*–*V* characteristics. (i) The correlation between the WF extracted from UPS results and the corresponding device *V*_OC_. The box plots in panels (d)–(g) display the mean, median line, 25%–75% box limits with 1.5× interquartile range whiskers. The total number of samples is 60. The corresponding values in panels (d)–(g) are provided in the Table S1.

As shown in Fig. 4a, the photoemission onset of samples with Pb-rich surfaces shifts by 0.4–0.5 eV as compared to the pristine sample. Consequently, the WF increases from 4.6 eV for the pristine sample to approximately 5 eV, corresponding to an upward shift of the vacuum level (Fig. 4b). Samples treated with Pb salts containing different halide anions resulted in similar WFs, suggesting the presence of an excess of Pb cation is more dominant in impacting the surface WF than the type of halide anion. CsPbI_3_ samples treated with methanol and DAB showed similar WF shifts to those treated with Pb salts, confirming that, regardless of the specific treatment process, a reduction in the surface Cs : Pb ratio is the main factor leading to an increase in the WF. The corresponding valence-band spectra show that the valence-band maximum (VBM) relative to the Fermi level for surfaces treated with PbI_2_, PbCl_2_, PbBr_2_, DAB, and methanol varies by only 0.25 eV. This indicates that the observed WF modifications are predominantly driven by surface dipole formation rather than by doping-induced Fermi-level shifts.

On the other hand, samples with Cs-rich surfaces exhibited a significant shift in the photoemission onset in the opposite direction. Consequently, the WF decreased from 4.6 eV for the pristine sample to 3.2 eV for a sample modified with 1 nm of CsBr. Notably, for the sample with 1 nm of RbI deposited on the CsPbI_3_ surface, the WF decreased even further to 2.9 eV (Fig. 4b). Interestingly, the samples treated with alkali metal salts show a substantial change in the spectral shape of the valence band, and a significant downward shift of the VBM (Fig. 4a). In these cases, the energy difference between the VBM and the Fermi level exceeds the bandgap of CsPbI_3_, suggesting the formation of wide-bandgap species at the film surface. The identification of the exact species that are formed is highly complex; for example, the CsI treatment might lead to the formation of Cs_1+*x*_PbI_3+*x*_ phases or unreacted CsI. This is consistent with the XPS measurements, which show a Cs/Pb ratio of 2.7, suggesting the presence of Cs-rich species. The exact composition of these species is difficult to ascertain, and the surface-sensitive nature of UPS makes it hard to probe the impact of their formation on the underlying CsPbI_3_ beyond the very significant change in WF.

The opposing trends in the WF shifts suggest that the modifications introduce surface dipoles of opposite orientation: Pb-rich surfaces tend to generate a positive dipole with negative charges oriented outward from the perovskite layer and positive charges oriented inward. In contrast, Cs- and Rb-rich surfaces generate a negative dipole in the opposite direction, as illustrated in Fig. 4c. A possible explanation for this observation is that for PbI_2_-rich surfaces, the Pb^2+^ ions from the Pb salt tend to form more stable, lower-energy [PbI_6_]^4−^ octahedra. This attracts I^−^ ions from the pristine perovskite surface *via* electrostatic and covalent interactions, causing electron density from within the perovskite to accumulate near the surface, thus forming a positive dipole. On Cs- or Rb-rich surfaces, halide ions introduced along with the alkali metal cations can coordinate with Pb^2+^ ions at the pristine perovskite surface, thereby filling I^−^ vacancies. This potentially leaves the alkali metal cations exposed, leading to the formation of a negative dipole.

To correlate the presence of such opposing surface dipoles with solar cell performance, we fabricated photovoltaic devices using all the above-mentioned surface treatments. Fig. 4d shows the distribution of solar cell PCEs, indicating that the efficiency of the devices can be essentially doubled from below 10% for devices with strong negative surface dipoles to ∼20% for devices with a positive dipole. This substantial variation in PCE is primarily driven by changes in *V*_OC_ (Fig. 4f) and FF (Fig. 4g), while the *J*_SC_ remains largely unaffected (Fig. 4e). The similarity between the evolutions in the *V*_OC_ and FF is consistent with the substantial changes in the built-in potential of the devices introduced by the surface dipoles: negative dipoles reduce the built-in potential, whereas positive dipoles enhance it.^41,42^ This is further confirmed by a direct comparison of the surface WF shift with *V*_OC_ (Fig. 4h), revealing a clear trend. Notably, the trend in the VBM position does not correlate with the measured *V*_OC_, further suggesting that the formation of surface dipoles, rather than other effects, is responsible for the observed changes in photovoltaic performance. This is also consistent with the fact that all modifications were introduced by only 1 nm, suggesting an abrupt change in energetics at the surface and consistent with the formation of a surface dipole.

Importantly, the choice of device architecture will play a crucial role in correlating surface dipole direction with device performance. Specifically, while negative dipoles are detrimental for PIN devices, they are expected to be beneficial for NIP devices, where a negative surface dipole would lead to enhanced built-in potential. Indeed, several literature reports have demonstrated that CsF and CsI treatments can enhance the performance of NIP devices, particularly by improving their *V*_OC_ and FF.^43,44^ This observation is further supported by a comparison of NIP and PIN devices with CsPbI_3_ active layers fabricated using the DMAI recipe without further surface treatment. Considering that such a recipe leads to an intrinsically CsI-rich CsPbI_3_ surface (Fig. 1c), it is to be expected that untreated layers would show a higher performance in the NIP – and not PIN – device structure. Literature reports are consistent with this, with untreated NIP structured devices often resulting in higher *V*_OC_ and FF than those of untreated PIN devices.^34,43,45,46^ These observations suggest that PIN devices are more likely to necessitate a surface treatment of the CsPbI_3_ active layer, and that treatments developed for NIP devices are ineffective in PIN structures.

We note that the different surface modifications impact on the hysteresis observed in the *J*–*V* curves of the devices. To quantify these changes, the hysteresis index for each device type was calculated and is listed in Table S1. Devices treated with PbI_2_, CsI, and RbI show increased hysteresis, which may be attributed to the introduction of additional iodide ions by these treatments, which could facilitate I^−^ ion migration and consequently increase hysteresis. In contrast, treatments with Br, Cl, and F salts did not lead to a notable change in hysteresis. We consider that this may be related to the stronger Pb–X (X = Br, Cl, F) bonding compared to Pb–I, which makes these ions less mobile, thereby mitigating the impact on hysteresis. Interestingly, treatments with DAB and methanol, which likely dissolve some CsI from the perovskite surface, had the opposite effect, reducing the hysteresis index. This is presumably because removing excess CsI reduces the source of mobile ions, thereby decreasing ion migration and the associated hysteresis.

### Impact of surface stoichiometry on device stability

**Fig. 5:**
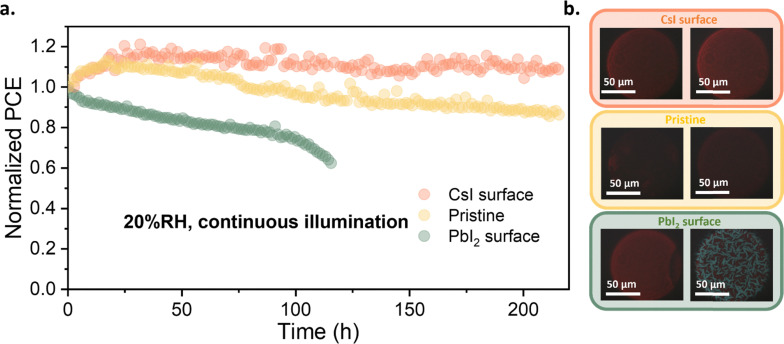
(a) Performance evolution of treated CsPbI_3_ solar cells under continuous illumination. Up to 250 h stability was tested under 20% relative humidity (RH), 25 °C (b) PL image of treated films before (left) and after stability (right) measurement. The image is presented in a true-color scale.

Next, we conducted stability tests on the devices under 20% relative humidity (RH) and AM1.5 light conditions (Fig. 5). The CsI surface device showed excellent stability, with no significant loss in performance after 220 hours, while the PCE of the pristine device decreased to ∼80% of its initial performance during the same time span. Interestingly, devices with a Pb-rich CsPbI_3_ surface, despite initially exhibiting substantially higher performance, degraded more rapidly, retaining only 60% of their initial performance after 120 hours (Fig. 5a). Thermal stability tests conducted at 60 °C (Fig. S6) show a similar trend to that observed under 1 sun illumination and 20% RH. PL microscopy (Fig. 5b) and XRD (Fig. S7) investigations reveal that this rapid degradation of samples with Pb-rich surface is related to a phase transition from the red-emitting β-CsPbI_3_ to the blue-emitting δ-CsPbI_3_. This could be associated with the face-sharing structure of [PbI_6_]^4−^ at the active layer surface, which, when in contact with β-CsPbI_3_ (which has a corner-sharing [PbI_6_]^4−^ structure), can trigger its transformation to the δ-CsPbI_3_ (which has an edge-sharing [PbI_6_]^4−^ structure), possibly enhanced by ion migration and illumination that are present during the stability measurements. In contrast, UPS experiments suggest the formation of wide-bandgap Cs-containing species at the surfaces of devices modified with CsI. These can, for example, follow a Cs_1+*x*_PbI_3+*x*_ structure, which contains partially isolated [PbI_6_]^4−^. Such a structure might suppress the formation of edge-sharing δ-CsPbI_3_, thereby enhancing the stability of the active layer. The relevant crystallographic structures are shown in Fig. S8.

## Discussion

The pronounced effect of surface stoichiometry on device performance in CsPbI_3_ solar cells is unlike that previously explored in other perovskite systems. For example, we have reported that tuning the surface stoichiometry of MAPbI_3_ layers can result in changes to their surface electronic structure, yet these changes are not associated with surface dipole formation since the WF of samples with different surface stoichiometries remained unchanged.^47^ Overstoichiometric MAPbI_3_ devices, although exhibiting higher device performance, also display lower photoluminescence responses,^48^ consistent with the presence of deep defects.^49^ Changing the stoichiometry of triple-cation perovskites has only a minor impact on their performance, with equally efficient devices fabricated across an extensive range of stoichiometries.^50^ Our results indicate that the surface of CsPbI_3_ is particularly sensitive to stoichiometric changes, with surface dipole formation that changes the surface WF across a range of >2 eV. The substantial modulation of the built-in potential through these surface dipoles dominates over any beneficial surface passivation effects and dictates the device performance. This highlights the need to systematically evaluate not only the efficacy of surface treatments in passivating surface defects of CsPbI_3_, but especially their effect on the surface stoichiometry and WF, thus making it possible to disentangle their individual impacts.

Moreover, the contrasting impact of surface stoichiometry on device performance and stability despite effective defect passivation suggests that the choice of device architecture is particularly crucial in the case of CsPbI_3_ perovskites. The performance of NIP devices, which benefit from the inherent Cs-rich nature of CsPbI_3_ fabricated *via* the DMAI method, can be further increased by methods that further increase the Cs : Pb surface ratio. As we showed above, this will not only introduce a negative dipole at the surface, leading to an increased built-in potential, but will also effectively passivate surface defects. Both these factors positively impact the devices’ performance. Our results show that the increase in the Cs : Pb ratio is also beneficial for improving the phase stability of CsPbI_3_, and consequently, that of the entire photovoltaic device.

PIN devices, on the other hand, require a surface treatment that will reduce Cs : Pb surface stoichiometry, leading to a positive dipole. Such treatments may also effectively passivate defects, resulting in an increased built-in potential and improved initial performance. However, additional measures to stabilize the black phase of CsPbI_3_ may be necessary in this case, as excess Pb at the perovskite surface could trigger its rapid degradation.

## Conclusions

In summary, this work demonstrates that adjusting the surface Cs : Pb ratio of CsPbI_3_ perovskites results in a substantial modulation in their surface WF and, consequently, the built-in potential of photovoltaic devices. While both Cs- and Pb-rich surfaces lead to effective defect passivation, device performance is dominated by the direction of the surface dipole and the chosen device architecture, meaning that surface treatments that passivate surface defects can be beneficial in one device architecture but detrimental in another. Importantly, we also demonstrate that surface stoichiometry has a significant impact on the stability of CsPbI_3_ layers, thereby highlighting that different device architectures may require distinct approaches for stability enhancement. These results highlight the crucial role that surface dipoles play in determining the performance and stability of CsPbI_3_ solar cells, thereby guiding device and interface design.

## Methods

Indium tin oxide (ITO)-coated glass substrates were bought from PsiOTech Ltd. (2-(3,6-Dimethoxy-9*H*-carbazol-9-yl)ethyl) phosphonic acid (MeO-2PACz), phenyl-C_61_-butyric acid methyl ester (PC_61_BM) (99.5%), and bathocuproine (BCP, 99.99%, trace metals basis) were purchased from TCI. All solvents were purchased from Sigma-Aldrich. For solution-processed CsPbI_3_, PbI_2_ (99.999%, trace metals basis), CsI (99.999%, trace metals basis) were obtained from TCI. The PbI_2_·*x*DMAI was prepared by using PbI_2_ (1.2 g) dissolved in 2.5 ml anhydrous dimethylformamide (DMF) at 80 °C under active stirring for 30 min in an air atmosphere. Immediately thereafter, 10 ml of hydroiodic acid was added to the solution, which was stirred for 24 h at 80 °C. The precipitate was filtered, then centrifuged and washed several times with copious diethyl ether and ethyl alcohol to remove the residual solvent. The collected powder was dried in a vacuum oven at 70 °C for 24 h. For evaporated metal halide salts, PbI_2_ (99.999%, trace metals basis), PbCl_2_ (99.999%, trace metals basis), PbBr_2_ (99.999%, trace metals basis), CsF (99.99%, trace metals basis), CsI (99.999%, trace metals basis) CsBr (99.999%, trace metals basis), and RbI (99.9%, trace metals basis), were obtained from Sigma-Aldrich. All materials were used as received without purification.

### Perovskite film deposition

Substrates for film deposition were ultrasonically cleaned with 2% Hellmanex detergent, deionized water, acetone, and isopropanol, followed by 15 min oxygen plasma treatment. In a dry air-filled glove box (RH < 1%), MeO-2PACz (3 mg ml^−1^ in ethanol) was spin-coated on the clean ITO substrates at 4000 rpm for 30 s and annealed at 100 °C for 10 min. The CsPbI_3_ precursor solution was prepared by dissolving 802.5 mg PbI_2_·*x*DMAI, 210.7 mg PbI_2_, and 415.9 mg CsI in 2 ml DMF and dimethyl sulfoxide (v/v, 9 : 1) under active stirring for 12 h at 60 °C. The CsPbI_3_ solution was spin-coated at 1000 rpm for 10 s and 4500 rpm for 30 s. The samples were then annealed at 180 °C for 15 min. Next, the samples were transferred into a physical vacuum deposition chamber (CreaPhys GmbH) to deposit 1 nm (obtained from QCM) of metal halide salts. Next, the samples were transferred into a nitrogen-filled glove box (GS), where PC_61_BM (20 mg ml^−1^ dissolved in chlorobenzene) was dynamically spin-coated at 2000 rpm for 30 s followed by a 3 min annealing at 100 °C. Finally, a BCP (0.5 mg ml^−1^ dissolved in isopropanol) hole-blocking layer was spin-coated at 4000 rpm for 30 s, followed by an 80 nm thermally evaporated Ag cathode (Mantis evaporator, base pressure of 10^−7^ mbar).

### Photovoltaic device characterisation

*J*–*V* characteristics of solar cells under a solar simulator (Abet Sun 3000 Class AAA solar simulator, AM 1.5 conditions) were recorded at room temperature in ambient conditions using a computer-controlled Keithley 2450 source meter unit. The incident light intensity was calibrated *via* a Si reference cell (NIST traceable, VLSI Standards Inc.) and tuned by measuring the spectral mismatch factor between the real solar spectrum, the spectral response of the reference cell, and the perovskite devices. All devices were scanned from short circuit to forward bias (1.2 V) and reversed with a rate of 0.025 V s^−1^. No treatment was applied before measurements. The active area for all devices was 4.5 mm^2^ defined by the overlap of thermally evaporated Ag and patterned ITO electrodes.

### SEM

An SEM (Gemini 500, ZEISS) with an acceleration voltage of 1.5 kV and a pressure of 5–6 × 10^−4^ mbar was employed to obtain surface and cross-sectional SEM images using the in-lens mode.

### XRD

XRD patterns were measured in ambient air using a Bruker Advance D8 diffractometer equipped with a 1.6 kW Cu-anode (*λ* = 1.54060 Å) and a LYNXEYE_XE_T 1D detector. The scans (2*θ*–*Ω* mode, 2*θ* = 10–40°, step size 0.01°, 0.1 s per step) were measured in a parallel beam geometry with a height-limiting slit of 0.2 mm. For grazing-incidence XRD, the parameters of scans are 2*θ* mode, 2*θ* = 10–15°, step size 0.01°, 0.5 s per step. The incidence angle (*Ω*) was fixed at 0.5°, 1°, 2°, 5°, respectively.

### UV-Vis absorption and PL measurement

The UV-vis absorbance spectra were recorded using a Shimadzu UV-3100 spectrometer. The wide-field PL images were measured using a home-built wide-field fluorescence microscope. The samples were excited using a 405 nm continuous wave laser (Pico Quant LDHDC375). A 40× dry objective lens (Olympus LUCPLFLN40X, NA = 0.6) was used for the excitation and collection of PL from the sample plane. After passing through the image lens in the emission path, the PL was imaged using a Thorlabs camera (CS895CU). PL measurement on complete solar cell devices were performed using a 532 nm laser inside an integrated sphere (Berlin/GermanyLP20-32) with spot size 1 cm^2^. A perovskite solar cell was used to adjust the laser intensity to 1 sun equivalent intensity, ensuring that the current density at 0 V was approximately equal to the *J*_SC_ obtained under standard solar simulator conditions.

### TRPL

A time-correlated single photon counting setup containing a 405 nm laser diode head (Pico Quant LDHDC375), a PMA Hybrid Detector (PMA Hybrid 40), a TimeHarp platine (all PicoQuant), and a Monochromator SpectraPro HRS-300 (Princeton Instruments) was utilized. Perovskite films on quartz were excited with the 405 nm laser diode. The emission was collected by the PMA hybrid detector.

### TA measurement

Femtosecond transient absorption experiments were carried out using a custom-built system. The primary light source is a 1 kHz Ti:Sapphire regenerative amplifier (Astrella-V, Coherent Inc.), which outputs 5 mJ pulses with a central wavelength of approximately 800 nm and a nominal pulse duration of 50 fs. A portion (*ca.* 18%) of the main beam was split off and employed for the generation of the 400 nm pump pulses *via* second harmonic generation, with an additional 2% split off and used to generate broadband probe light *via* bulk supercontinuum generation in sapphire. The resulting supercontinuum was split into separate probe and reference beams. The probe beam was overlapped with the 400 nm pump beam in the sample, after which it was directed to a fibre-coupled imaging spectrometer (UV-2048-CL-EVO, Avantes BV). The reference beam was also collimated and directed into an identical fibre coupled spectrometer. The size of the probe spot at the sample plane was approximately 0.1–0.2 mm; to ensure even illumination over the probed region of the material, the probe spot was kept at approximately 0.6–0.8 mm. The pump fluence was controlled using a variable attenuator and kept at a value of 1.8 µJ cm^−2^.

The pump probe-delay was controlled using a motorized delay line (DDS600M, Thorlabs Inc). Pump–probe delay times ranged from −30 ps to 3 ns, with time points distributed logarithmically on both sides of time zero to capture the rapid evolution of carrier dynamics shortly following excitation. For each delay time, spectra were averaged over 600 individual laser shots. To correct for spatial inhomogeneities in the perovskite films, a total of 200 different points on each system were sampled, and the resulting TA maps averaged. Tabulated lifetimes of the TA probed kinetics shown in Fig. 3e are listed in Table S2.

### XPS and UPS

The samples were transferred to an ultrahigh vacuum chamber (ESCALAB 250Xi, Thermo Scientific) with a base pressure of 2 × 10^−10^ mbar for XPS and UPS measurements. In the XPS measurements, an X-ray beam is generated by an XR6 monochromated Al Kα source (*hν* = 1486.6 eV) with a pass energy of 20 eV. UPS measurements were performed using a double differentially pumped He gas discharge lamp emitting He I radiation (*hν* = 21.22 eV) with a pass energy of 2 eV and a bias of −5 V to ensure secondary electron onset detection.

## Author contributions

Conceptualization: R. J., Y. V.; data curation: R. J., N. G.; investigation: R. J.; methodology: R. J., N. G., S. S., R. B., Y. D., Z. Z., F. K., M. D., V. S., J. R. B.-Q.; writing – original draft: R. J., Y. V.; writing – review & editing: R. J., N. G., S. S., R. B., Y. D., Z. Z., F. K., M. D., V. S., J. R. B.-Q., B. R., Y. V.; supervision: Y. V.

## Conflicts of interest

There are no conflicts to declare.

## Supplementary Material

EE-019-D5EE07787G-s001

## Data Availability

The data supporting this article have been included as part of the supplementary information (SI), including the related measurements, such as the spectroscopic and crystallographic characterisations. See DOI: https://doi.org/10.1039/d5ee07787g.
