# Supplementary material for: The critical role of surface dipoles in CsPbI3 perovskite solar cells
Source: Energy Environ Sci. 2026 Mar 11;19(9):2863–73. doi: 10.1039/d5ee07787g (PMC13014367; doi:10.1039/d5ee07787g)
Supplement: EE-019-D5EE07787G-s001 [file EE-019-D5EE07787G-s001.pdf]

Supplementary Information (SI) for Energy & Environmental Science.
This journal is © The Royal Society of Chemistry 2026

Supporting Information

The Critical Role of Surface Dipoles in $CsPbI_3$ Perovskite Solar Cells

*Ran Ji, Nathaniel Gallop, Shivam Singh, Richard Beier, Yitian Du, Zongbao Zhang, Fulya Koc, Marielle Deconinck, Vladimir Shilovskikh, Jose Roberto Bautista-Quijano, Boris Rivkin, and Yana Vaynzof**

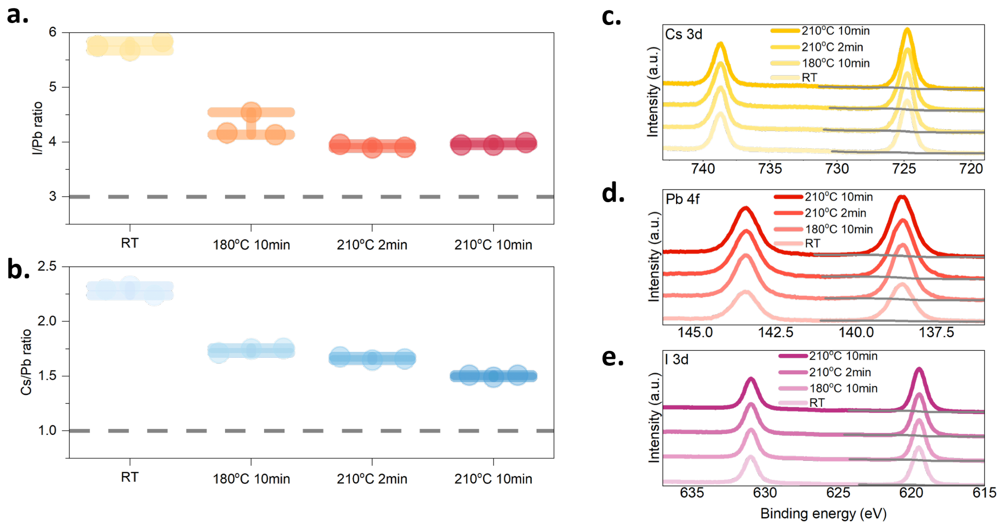

Figure S1 a. I/Pb, and b. Cs/Pb element ratio distribution of $CsPbI_3$ films with different annealing conditions. The values were obtained from XPS measurements, with three different areas tested under each condition to ensure repeatability. Representative XPS spectra are shown in c. (Cs 3d), d. (Pb 4f), and e. (I 3d).

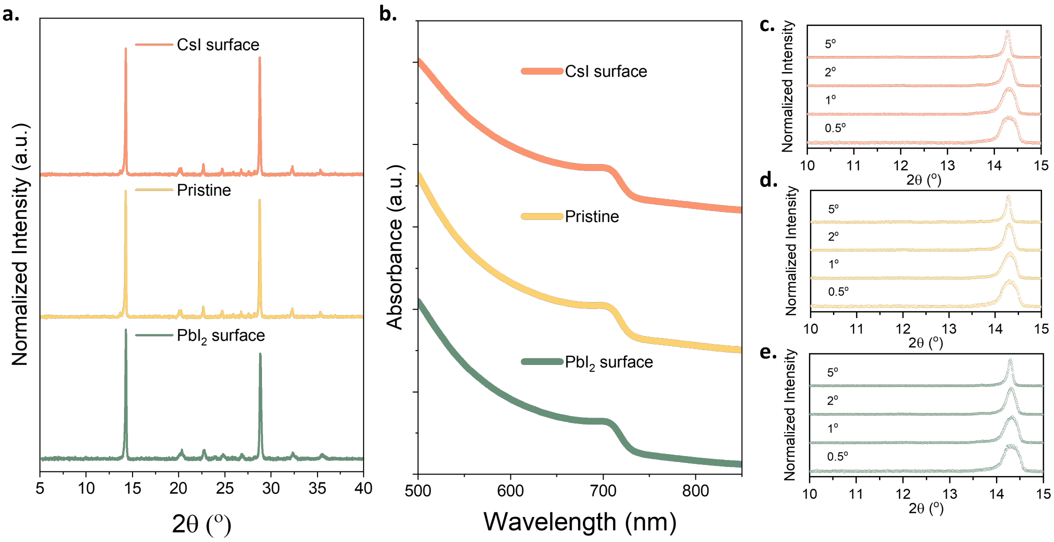

Figure S2 a. XRD patterns, b. UV-Vis absorption spectra, and patterns of $CsPbI_3$ films with varied surface conditions.

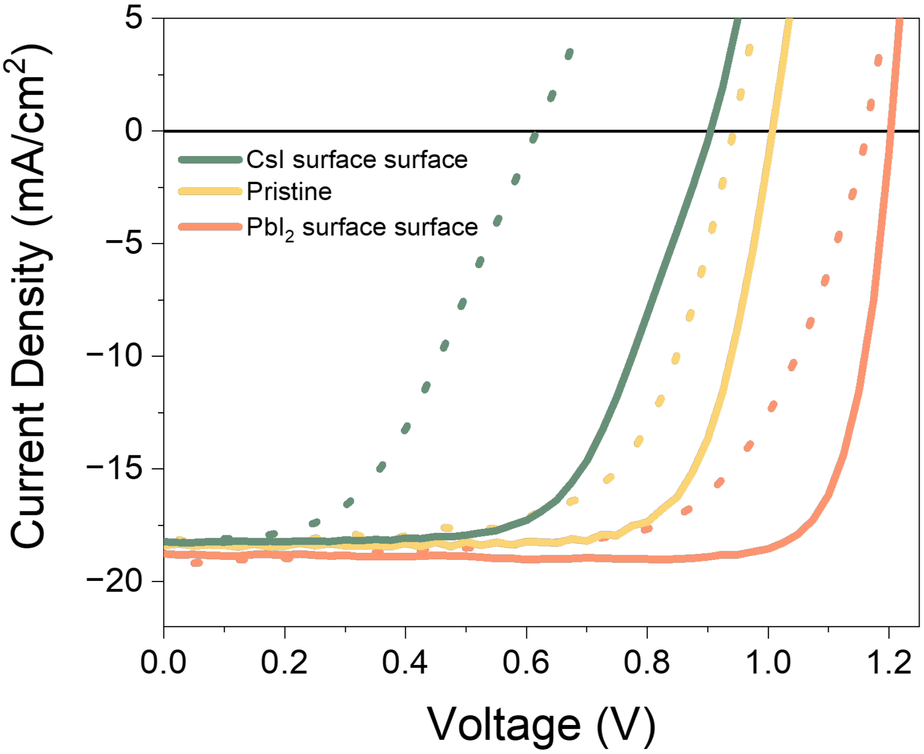

Figure S3 J-V characteristics of Figure 2f measured with forward and reverse scans. The dashed line represents the forward scan, and the solid line represents the reverse scan.

Table S1 PV parameters of solar cells with treated-$CsPbI_3$ active layers in Figure 4.

	V_{OC} (V) Forward	J_{SC} (mA/cm^2) Forward	FF (%) Forward	PCE (%) Forward	V_{OC} (V) Backward	J_{SC} (mA/cm^2) Backward	FF (%) Backward	PCE (%) Backward	Maximum PCE (%)	Hysteresis Index
PbI_2	1.16±0.02	-19.81±0.60	59.58±8.37	13.63±1.47	1.19±0.01	-18.66±0.65	82.98±0.31	18.44±0.61	19.17	0.26±0.15
$PbBr_2$	1.16±0.01	-19.52±0.88	70.22±4.49	15.90±0.85	1.18±0.01	-19.31±0.65	82.00±0.16	18.76±0.66	19.73	0.15±0.11
$PbCl_2$	1.15±0.04	-19.02±1.05	71.37±4.88	15.63±0.82	1.19±0.01	-18.95±0.97	81.56±0.09	18.40±0.98	19.60	0.15±0.12
DAB	1.13±0.02	-19.31±0.42	72.09±7.07	15.64±1.25	1.15±0.00	-18.95±0.33	80.73±1.51	17.62±0.42	18.20	0.11±0.13
Methnol	1.10±0.04	-18.95±0.25	70.34±5.74	14.72±1.28	1.14±0.00	-18.68±0.51	79.66±1.39	17.02±0.52	17.57	0.14±0.19
Pristine	0.95±0.04	-18.88±0.63	64.53±2.23	11.55±0.52	1.00±0.01	-18.84±0.52	72.26±1.64	13.68±0.39	14.31	0.15±0.14
CsF	0.87±0.10	-17.66±0.75	58.36±3.17	9.00±1.48	0.93±0.01	-17.66±0.75	64.67±2.46	10.60±0.83	11.60	0.15±0.34
CsI	0.80±0.10	-18.63±0.82	55.20±5.22	8.37±1.90	0.90±0.00	-18.63±0.82	63.35±1.10	10.65±0.47	11.53	0.22±0.47
CsBr	0.79±0.05	-18.31±1.00	53.81±5.44	7.85±1.54	0.84±0.01	-18.31±1.00	59.05±3.47	9.09±0.76	10.09	0.14±0.28
RbI	0.68±0.10	-18.24±0.44	52.74±10.25	6.68±2.11	0.78±0.01	-18.24±0.44	63.05±3.50	8.98±0.68	10.29	0.26±0.51

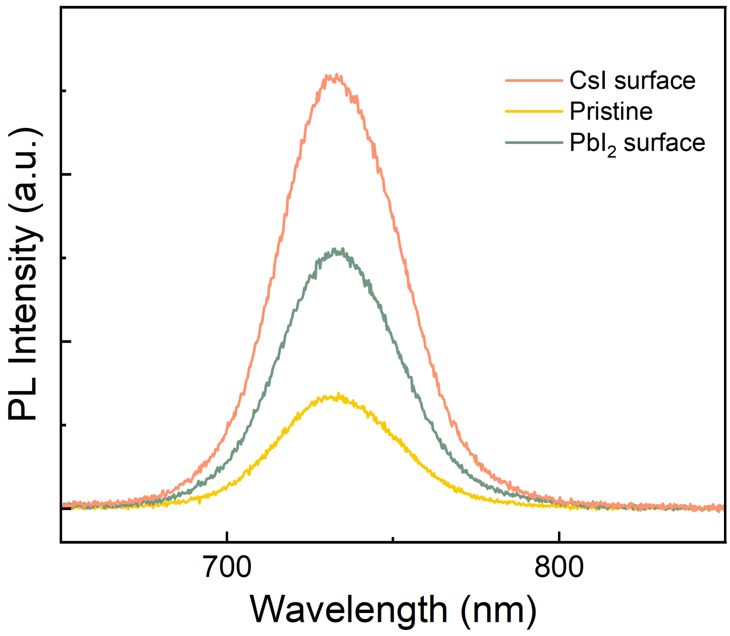

Figure S4 1-sun steady-state photoluminescence (PL) spectra of complete $CsPbI_3$ perovskite solar cells with architecture of ITO/MeO-2PACz/$CsPbI_3$/PCBM/BCP/Ag.

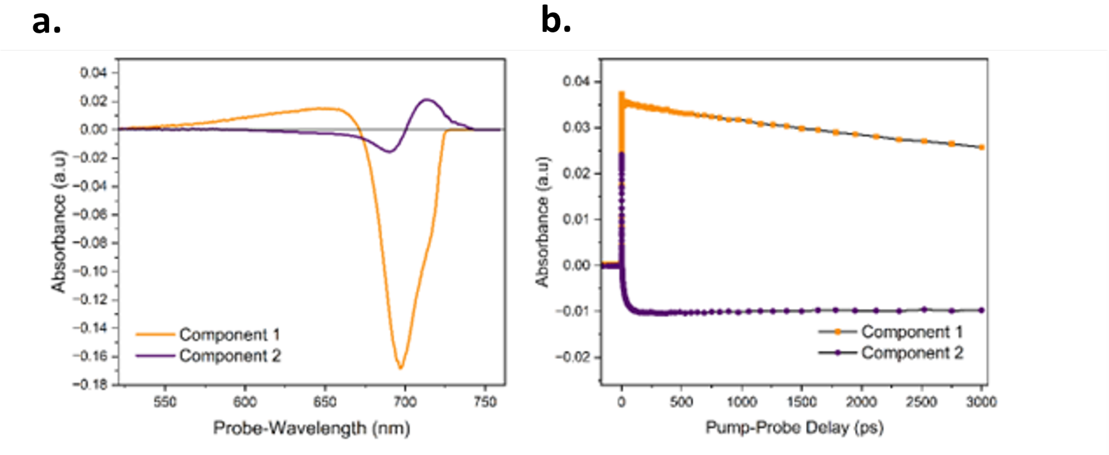

Figure S5 a. component-spectra and b. associated dynamics retrieved from the Singular Value Decomposition of $CsPbI_3/PbI_2$/PCBM TAS-data given in Figure 3. Component 1 incorporates the negative GSB-signal at 700nm alongside a broad photoinduced absorption signal around 665 nm. The spectral shape and kinetics of component 2 are consistent with energetic redistribution processes arising from hot-carrier and band-filling effects.

Table S2 Tabulated lifetimes of the kinetics given in Figure 3e.

System	Average Lifetime (ns)		Change in Lifetime (ns)
	Bare System (No PCBM)	Interfaced with PCBM	
**$CsPbI_3$**	8.03 ± 0.1	5.41 ± 0.21	2.62 ± 0.31
**PbI_2 surface**	11.08 ± 0.18	6.96 ± 0.24	4.12 ± 0.44
**CsI surface**	9.62 ± 0.13	8.56 ± 0.19	1.06 ± 0.32

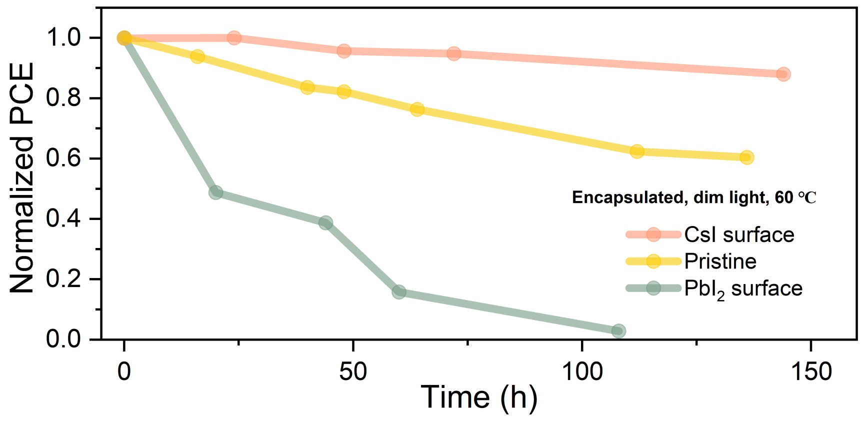

Figure S6. Performance evolution of treated $CsPbI_3$ solar cells with encapsulation. Up to 150 h stability was tested under dim light, 60 °C

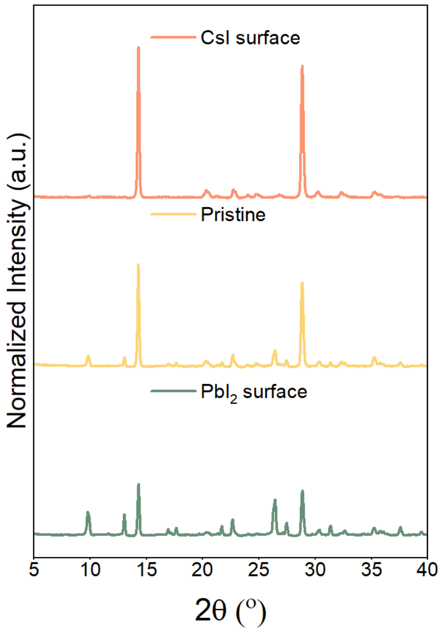

Figure S7 XRD patterns of treated $CsPbI_3$ films after degradation. The condition is maintained under continuous illumination for up to 250 h at 20% relative humidity (RH) and 25 °C.

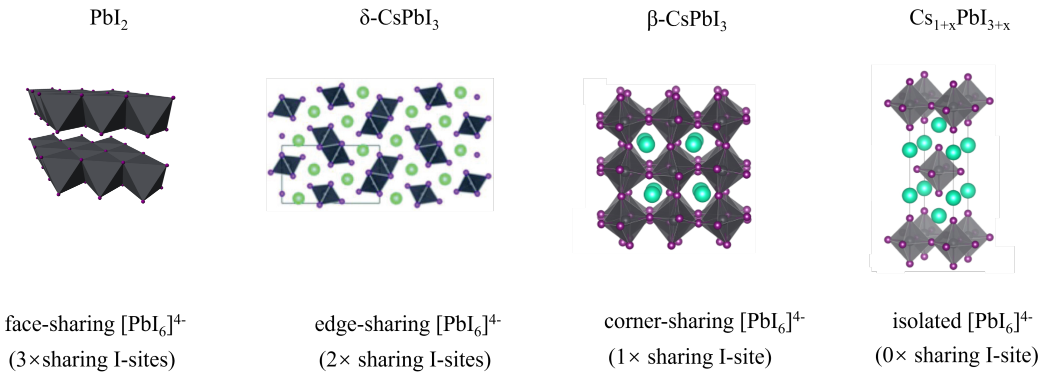

Figure S8 Schmetic diagram of the crystallographic structure of PbI_2, δ-$CsPbI_3$, β-$CsPbI_3$, and $Cs_{1+x}PbI_{3+x}$.
